# Sleep and Neurochemical Modulation by DZNep and GSK-J1: Potential Link With Histone Methylation Status

**DOI:** 10.3389/fnins.2019.00237

**Published:** 2019-03-15

**Authors:** Eric Murillo-Rodríguez, Gloria Arankowsky-Sandoval, Jorge Aparecido Barros, Nuno Barbosa Rocha, Tetsuya Yamamoto, Sérgio Machado, Henning Budde, Diogo Telles-Correia, Diogo Monteiro, Luis Cid, André Barciela Veras

**Affiliations:** ^1^Laboratorio de Neurociencias Moleculares e Integrativas, Escuela de Medicina División Ciencias de la Salud, Universidad Anáhuac Mayab, Mérida, Mexico; ^2^Intercontinental Neuroscience Research Group, Mérida, Mexico; ^3^Centro de Investigaciones Regionales “Dr. Hideyo Noguchi” Universidad Autónoma de Yucatán, Mérida, Mexico; ^4^Post-graduation Program of Psychology of Health, NACNeuro, Dom Bosco Catholic University, Campo Grande, Mato Grosso del Sur, Brazil; ^5^School of Health, Polytechnic Institute of Porto, Porto, Portugal; ^6^Graduate School of Technology, Industrial and Social Sciences, Tokushima University, Tokushima, Japan; ^7^Laboratory of Physical Activity Neuroscience, Physical Activity Sciences Postgraduate Program, Salgado de Oliveira University, Niterói, Brazil; ^8^Faculty of Human Sciences, Medical School Hamburg, Hamburg, Germany; ^9^Institute of Sport Science and Innovations, Lithuanian Sports University, Kaunas, Lithuania; ^10^University of Lisbon, Faculty of Medicine, Lisbon, Portugal; ^11^Sport Science School of Rio Maior- Polytechnic Institute of Santarém, Rio Maior, Portugal; ^12^Research Center in Sport, Health and Human Development-CIDESD, Vila Real, Portugal

**Keywords:** adenosine, dopamine, histone demethylation, serotonin, sleep, wakefulness

## Abstract

Histone methylation/demethylation plays an important modulatory role in chromatin restructuring, RNA transcription and is essential for controlling a plethora of biological processes. Due to many human diseases have been related to histone methylation/demethylation, several compounds such as 3-deazaneplanocin A (DZNep) or 3-((6-(4,5-Dihydro-1H-benzo[d]azepin-3(2H)-yl)-2-(pyridin-2-yl)pyrimidin-4-yl)amino)propanoic acid; *N*-[2-(2-pyridinyl)-6-(1,2,4,5-tetrahydro-3H-3-benzazepin-3-yl)-4-pyrimidinyl]-β-Alanine (GSK-J1), have been designed to inhibit histone methylase or suppress histone demethylase, respectively. In the present study, we investigated the effects on the sleep-wake cycle and sleep-related neurochemical levels after systemic injections of DZNep or GSK-J1 given during the light or dark phase in rats. DZNep dose-dependently (0.1, 1.0, or 10 mg/kg, i.p.) prolonged wakefulness (W) duration while decreased slow wave sleep (SWS) and rapid eye movement sleep (REMS) time spent during the lights-on period with no changes observed in dark phase. In opposite direction, GSK-J1 (0.1, 1.0, or 10 mg/kg, i.p.) injected at the beginning of the lights-on period induced no statistical changes in W, SWS, or REMS whereas if administered at darkness, we found a diminution in W and an enhancement in SWS and REMS. Finally, brain microdialysis experiments in freely moving animals were used to evaluate the effects of DZNep or GSK-J1 treatments on contents of sleep-related neurochemicals. The results showed that DZNep boosted extracellular levels of dopamine, norepinephrine, epinephrine, serotonin, adenosine, and acetylcholine if injected at the beginning of the lights-on period whereas GSK-J1 exerted similar outcomes but when administered at darkness. In summary, DZNep and GSK-J1 may control the sleep-wake cycle and sleep-related neurochemicals through histone methylation/demethylation activity.

## Introduction

DNA methylation and modifications of histones are part of molecular mechanisms that control gene expression promoting the stabilization of the genome as well (Almeida et al., 2017; Bauer and Martin, 2017; Kim and Kaang, 2017; Pasculli et al., 2018). In the recent years, multiple studies have demonstrated the relationship between histone methylation/demethylation activity and various human pathologies, such as cancer (Zaidi et al., 2017; Zam and Khadour, 2017). Understanding the molecular mechanism by which histone methylation/demethylation is translated into molecular modulators is essential to developing medicinal approaches for disease genetics and genome-related medicine.

This research area is complex, but there is an increasing appreciation of the role of histones in health and disease. Current experimental pharmacological approaches for treating histone-related disorders are based on designed drugs aimed to manage genetic-related health disturbances (Li et al., 2016; Boehm and Ott, 2017; McCabe et al., 2017; Kaniskan et al., 2018; Khan and Haqqi, 2018; Thomas and D’Mello, 2018). Converging studies have suggested that compounds such as 3-deazaneplanocin A (DZNep), inhibits histone trimethylation of lysine 27 on histone H3 and lysine 20 on histone H4 whereas treatments with 3-((6-(4,5-Dihydro-1H-benzo[d]azepin-3(2H)-yl)-2-(pyridin-2-yl)pyrimidin-4-yl)amino)propanoic acid; *N*-[2-(2-pyridinyl)-6-(1,2,4,5-tetrahydro-3H-3-benzazepin-3-yl)-4-pyrimidinyl]-β-Alanine (GSK-J1), the histone demethylase activity is suppressed (Dagdemir et al., 2016; Kuntz et al., 2016; Kaniskan et al., 2018; Zheng et al., 2018).

The histone methylation/demethylation activity is a promising target for exploring therapeutical options to treating multiple disorders. More recently, it has been demonstrated the role of histones in neurodegenerative diseases, aging, learning and memory (Jêśko et al., 2017; Kaluski et al., 2017; Zhao et al., 2018). Thus, the rationale use of histone methylation/demethylation inhibitors represents potential utility of DZNep or GSK-J1 in the comprehension of the role of histones in neurobiological processes, such as the sleep-wake cycle. In this report, we investigated the behavioral and neurochemical consequences that result from systemic injections of DZNep or GSK-J1, specifically in the sleep-wake cycle and extracellular levels of monoamines, adenosine and acetylcholine in rats.

## Materials and Methods

### Experiment 1: Effects on Sleep After Injections of DZNep or GSK-J1 During the Lights-On or the Lights-Off Period

#### Ethics Statement

The whole experimental procedures were approved by the Research and Ethics Committee of our Institution fulfilling the Mexican Standards Related to Use and Management of Laboratory Animals (DOF. NOM-062-Z00-1999), the U.K. Animals Scientific Procedures (Act, 1986 and associated guidelines, EU Directive 2010/63/EU for animal experiments), National Institute of Health (NIH Publication No. 80-23, revised 1996 and Guide for the Care and Use of Laboratory Animals, 8th Edn, 2011), and ARRIVE standards of Animal Welfare. For ethical reasons, efforts to minimize animal suffering were considered during the whole experiment and a reduced number of animals were included in the current report.

#### Animals

Male Wistar rats (*n* = 20; 250–300 g) were singly housed in polycarbonate cages (48.26 cm × 26.67 cm × 20.32 cm; Harlan Laboratories, Mexico) under humidity (60 ± 10%), ambient temperature (21 ± 1°C) and light–dark cycle 12:12 (lights-on: 07:00–19:00 h) controlled. All rats had free access to Purina Rat Chow (Purina, Mexico) as well as tap water.

#### Chemical

Chemical compounds studied in this article DZNep (PubChem CID: CID: 73087); GSK-J1 (PubChem CID: 56963315); polyethylene glycol (PubChem CID: 174). More information is available at https://pubchem.ncbi.nlm.nih.gov/. DZNep and GSK-J1 were purchased from Sigma (St. Louis, MO, United States) and dissolved in a vehicle (VEH) solution composed of polyethylene glycol/saline (5:95 v/v) as previously reported (Miranda et al., 2009; Johansson et al., 2016). Additional reagents, chemicals, and materials were purchased from Sigma-Aldrich (St. Louis, MO, United States) or Bioanalytical Systems (West Lafayette, IN, United States).

#### Sleep-Recording Surgeries

Anesthesia (acepromazine [0.75 mg/kg], xylazine [2.5 mg/kg], and ketamine [22 mg/kg]) was given (i.p.) to rats to place them in a stereotaxic frame (David Kopf Instruments, Tujunga, CA, United States) for sleep-recording electrodes surgery. Briefly, two stainless-steel screw electrodes were placed 2 mm on either side of the sagittal sinus and 3 mm anterior to Bregma (frontal cortex). Other two screws were located 3 mm on either side of the sagittal sinus and 6 mm behind Bregma (occipital cortex). The whole electrodes recorded the electroencephalogram (EEG) activity by the bipolar (differential) EEG recorded from the two contralateral screw electrodes (frontal-occipital). The electromyogram (EMG) activity was obtained by the implantation of two wire electrodes into the dorsal neck muscles. Finishing the EEG/EMG electrode implantation, the wires were inserted into a six-pin plastic plug (Plastics One, Roanoke, VA, United States) and attached onto the skull by dental cement. Upon completion of the EEG/EMG electrodes surgeries, animals were placed into individual cages with food and water *ad libitum*. In addition, rats were connected to a 6-channel slip-ring commutator through a 50 cm cable (Plastics One, Roanoke, VA, United States), which allowed free moving of animal around the cage. The EEG/EMG electrodes surgical procedure as well as habituation conditions were carried out as reported previously (Murillo-Rodríguez et al., 2017).

#### Pharmacological Administrations

Right after the surgeries, animals spent seven continuous days for recovery from EEG/EMG electrodes surgery as well as for habituation to the experimental conditions. Once completed this period, animals were placed randomly into one of the experimental blocks (either lights-on or lights-off period): Control ([CTL] VEH; *n* = 5), DZNep (0.1, 1.0, or 10 mg/kg, i.p.; each dose, *n* = 5) or GSK-J1 (0.1, 1.0, or 10 mg/kg, i.p.; each dose, *n* = 5). Doses of compounds were chosen arbitrarily since no direct evidence was available regarding their effects on freely moving rats. Moreover, the route of administration of compounds was selected to obtain a preliminary approach for an understanding of the putative phenomena. To avoid circadian influences in the pharmacological treatments of the histone methylation/demethylation activity on sleep, the administrations were done 1 h after either the start of the lights-on or the lights-off period. Thus, animals were disconnected from the sleep-recording system and experimental trials were administered. Once experimental challenges were applied, rats were reattached to the sleep-recording system and sleep data were collected across the next 4 h. Lastly, due to the reduced number of animals used for ethical reasons, the experiment was under a single-blind Latin Square Experimental Design. Since each subject serves as its own control, the testing paradigm has the advantage of decreasing variability among experimental conditions.

#### Analysis of Sleep Recordings

The sleep data were collected during the following 4 h post-injections sampled in periods of 12 s (epochs). Next, differentiation of wakefulness (W), slow wave sleep (SWS), or rapid eye movement sleep (REMS) was based on the characterization of each phase by the aid of the sleep-scoring program (ICELUS) as previously reported (Murillo-Rodríguez et al., 2017). One observer blind to the experiments analyzed sleep recordings.

#### Power Spectra Analysis

Fast Fourier transformation analysis for alpha (across W [α: 12–16 Hz]), delta (for SWS [Δ: 0.3–4.0 Hz]), and theta (during REMS [Θ: 6.0–12.0 Hz]) was collected during either lights-on or lights-off after experimental trials. This data provided information about quality rather than quantity of the sleep stages under the influence of treatments. Data were analyzed as previously reported (Murillo-Rodríguez et al., 2017). One observer blind to the experiments analyzed power spectra.

#### Statistical Analysis

The data for the experimental results were presented as mean ± SEM. The data were examined by ANOVA test followed by Scheffé’s *post hoc* test using the StatView software (version 5.0.0, SAS Institute, United States). Statistical differences among groups were determined if *P* < 0.05.

### Experiment 2: Effects on the Extracellular Levels of Monoamines After Administrations of DZNep or GSK-J1

#### Animals

A new set of male Wistar rats (*n* = 20; 250–300 g) were maintained as described in Experiment 1.

#### Chemicals

As described in Experiment 1.

#### Microdialysis Surgeries

Previous results have shown the role of the nucleus accumbens (AcbC) in the sleep-wake cycle modulation (Qiu et al., 2012). Moreover, studies from our group have demonstrated reliable measurements of sleep-related neurochemicals such as dopamine (DA), norepinephrine (NE), epinephrine (EP), serotonin (5-HT), of samples collected from AcbC (Mijangos-Moreno et al., 2014, 2016; Murillo-Rodríguez et al., 2017). Thus, we determined whether DZNep or GSK-J1 might exert changes in the levels of these neurochemicals as characterized from microdialysis samples collected from AcbC. To achieve this goal, animals were anesthetized and mounted into the stereotaxic frame (David Kopf Instruments, Tujunga, CA, United States) for implantation of a microdialysis guide-cannula (IC guide; BioAnalytical Systems [BAS], West Lafayette, IN, United States) aimed unilaterally into the AcbC (coordinates: A = +1.2, L = +2.0, and H = −7.0 mm, with reference to Bregma [Paxinos and Watson, 2005]). Right after the surgery, rats were placed individually into the microdialysis bowl (Raturn Microdialysis Stand-Alone System, MD-1404, BAS, West Lafayette, IN, United States) for post-surgery recovery (seven continuous days) as well as habituation for the experimental conditions. All surgical procedures of microdialysis surgery were accomplished as previously reported (Murillo-Rodríguez et al., 2017).

#### Pharmacological Administrations

As described in Experiment 1.

#### Microdialysis Sampling Procedures

Once reaching the recovery and habituation time, animals were removed from microdialysis bowls and the stylet from guide-cannula was withdrawn. Next, the microdialysis probe (1 mm of length; polyacrylonitrile, MWCO = 30,000 Da; 340 μm OD; BAS, West Lafayette, IN, United States) was inserted at 07:00 h. Artificial cerebrospinal fluid (aCSF [composition: NaCl 147 mM, KCl 3 mM, CaCl 1.2 mM, MgCl 1.0 mM, pH 7.2]) was perfused through a miniature tube (0.65 mm OD × 0.12 mm ID; BAS, West Lafayette, IN, United States) attached to a 2.5 mL syringe (BAS, West Lafayette, IN, United States) with a pump (flow rate: 0.25 μL/min; BAS Bee, West Lafayette, IN, United States). As standardized procedure, before the beginning of the experiments, in all rats, the microdialysis membrane was stabilized during 24 h and samples from that period were excluded for the final analysis as suggested from previous reports (Mijangos-Moreno et al., 2014, 2016; Murillo-Rodríguez et al., 2017). Right after the period of stabilization of membrane was covered, experimental trials were given and animals were reattached to the microdialysis system. The dialysates were collected every 20 min at the beginning of each hour during the following 4 h. Later, all samples were stored (−80°C) for further analysis. The microdialysis sampling procedure was developed as previous reports (Mijangos-Moreno et al., 2014, 2016; Murillo-Rodríguez et al., 2017).

#### Neurochemical Analysis of Monoamines

Dialysates collected from sampling were filtered (Millipore 0.22 μm; Merck Millipore, Darmstadt, Germany) and injected into high performance liquid chromatography (HPLC; Modular Prominence, Shimadzu, Kyoto, Japan). For monoamines detection, the HPLC used mobile phase (monosodium phosphate) ([7 mM, pH 3.0], plus methanol [3.5%]) which was perfused at flow rate of 80 μL/min (pump LC-20AT, Shimadzu, Kyoto, Japan). Separation of molecules was achieved by using a microbore column (octadecyl silica [3 μm, 100 mm × 1 mm], BAS, West Lafayette, IN, United States) with temperature controlled (22°C; oven CTO-20A, Shimadzu, Kyoto, Japan). The detection of monoamines was obtained by using an electrochemical detector (LC-4C; BAS, West Lafayette, IN, United States). The chromatographic data were obtained and stored on a personal computer (via computer controller CBM-20A, Shimadzu, Kyoto, Japan). The concentrations of monoamines in the samples were obtained by comparing external known standards for the neurochemicals studied (DA, NE, EP, and 5-HT) injected into HPLC. The whole HPLC procedures were developed as described previously (Murillo-Rodríguez et al., 2017). One observer blind to the code of experimental samples analyzed contents of monoamines.

#### Statistical Analysis

The total values for monoamines contents consisted in samples summed from continuous 4 h. All data from microdialysis experiments were represented as mean ± SEM. Statistical differences among the experimental groups were determined by one-way ANOVA followed by Scheffé’s *post hoc* test for multiple comparisons. All statistical analyses were performed using the StatView software (version 5.0.0, SAS Institute, United States) and statistical differences among groups were determined if *P* < 0.05.

### Experiment 3: Effects on the Extracellular Contents of Adenosine After Treatments of DZNep or GSK-J1

#### Animals

As described in Experiment 2.

#### Chemicals

As described in Experiment 2.

#### Microdialysis Surgeries

As described in Experiment 2.

#### Administration of Drugs

As described in Experiment 1.

#### Microdialysis Sampling Method

As described in Experiment 2.

#### Neurochemical Analysis of Adenosine

Similar chromatographic conditions as described for detection of monoamines were used for adenosine (AD) analysis. Methodological differences consisted in using an UV detector (SPD-20A Prominence, Shimadzu, Kyoto, Japan) setting the wavelength at 254 nm (deuterium lamp) and mobile phase (10 mM sodium dihydrogen phosphate [pH 4.5] and methanol [9%]) which was perfused at a flow rate of 80 μL/min using a pump (LC-20AT, Prominence HPLC, Shimadzu, Kyoto, Japan). All chromatographic data for AD detection were recorded in a personal computer, and peak heights of this purine in dialysates were compared with external known standards by using chromatograph report software (LC Solution, Shimadzu, Kyoto, Japan). The whole procedure for detection and measurement of AD using HPLC means was developed according to previous reports (Mijangos-Moreno et al., 2014; Murillo-Rodríguez et al., 2017). Contents of AD were analyzed under a blind experimental design.

#### Statistical Analysis

As described in Experiment 2.

### Experiment 4: Effects on the Extracellular Contents of Acetylcholine After Administrations of DZNep or GSK-J1

#### Animals

A new set of male Wistar rats (*n* = 20; 250–300 g) were used as described in Experiment 2.

#### Chemicals

As described in Experiment 1.

#### Microdialysis Surgeries

The importance of basal forebrain in the sleep-wake modulation has been previously reported (Anaclet et al., 2015; Gold et al., 2015; Xu et al., 2015). Moreover, it has been demonstrated that acetylcholine (ACh) is measurable from the mentioned brain area (Vanini et al., 2012; Zaghloul et al., 2017). Then, in this part of the study, we placed the microdialysis probe into the basal forebrain following stereotaxic coordinates (A = −0.35; L = 2.0; and H = −7.5 mm, with reference to Bregma [Paxinos and Watson, 2005]). All microdialysis surgery procedure was developed as described in Experiment 2.

#### Pharmacological Administrations

As described in Experiment 2.

#### Microdialysis Sampling Procedures

As described in Experiment 2 with exception of aCSF (KCl [2.4 nM], Na_2_SO_4_ [0.5 nM], NaCl [126.5 nM], CaCl_2_ [1.2 nM], NaHCO_3_ [27.5 nM], KH_2_PO_4_ [0.5 nM], MgCl_2_ [0.8 nM], dextrose [5.0 nM] at pH [6.8 ± 0.1]) which was perfused through a miniature tubing (0.65 mm OD × 0.12 mm ID; BAS, West Lafayette, IN, United States) attached to a 2.5 mL syringe (BAS, West Lafayette, IN, United States) with a pump (flow rate: 2 μL/min; BAS Bee, West Lafayette, IN, United States).

#### Analysis of Contents of Acetylcholine Using the HPLC

As described in Experiment 2 with exception of conditions for ACh detection. In detail: neostigmine bromide (100 nM) was added to the aCSF to facilitate detection of ACh contents. Moreover, the use of acetylcholine-choline assay kit (MF-8910; BAS, West Lafayette, IN, United States) was included for detection of ACh. Next, for separation of molecule, samples were injected into the HPLC at a flow rate of 1 mL/min, with temperature of 28°C (oven CTO-20A, Shimadzu, Kyoto, Japan), on 10 cm analytical column (MF-6150, BAS, West Lafayette, IN, United States) by using a mobile phase (Na_2_HPO_4_ [35 nM], EDTA [0.1 nM], and ProClin 150 preservative (0.005%), BAS, West Lafayette, IN, United States), and adjusted to pH 8.5 with phosphoric acid. By electrochemical means (LC-4C; BAS, West Lafayette, IN, United States) and maintaining the potential of +0.5 V, ACh was detected in collected samples. The analytical procedure to determinate ACh was developed as previous reports (Grupe et al., 2013; Murillo-Rodríguez et al., 2018). Extracellular levels of ACh were analyzed under a blind experimental design.

#### Recovery *in vitro* for Calibration of Microdialysis Probes

In the whole microdialysis experiments, measuring monoamines, AD or ACh, the microdialysis probes were used no more than five continuous days since previous reports have shown that the permanence of the cannula in the study area for more than 5 days diminishes the membrane ability to transport fluid through the microdialysis probe pores (Porkka-Heiskanen et al., 2000; Murillo-Rodríguez et al., 2004). In addition, we developed recovery test of microdialysis cannula by inserting the probes into a test solution containing external known concentrations of monoamines, AD or ACh under controlled perfusion of respective mobile phase. Dialysates were collected in a triplicate fashion under these experimental conditions. Each sample collected from *in vitro* recovery study was analyzed and the peak area ratio for monoamines, AD or ACh was calculated against the known standards. For obtaining the recovery rate, data were calculated by using the formulae: recovery rate (%) D (the peak area ratio of the sample from microdialysis sample)/(the peak area ratio of the sample in the test solution). The whole recovery *in vitro* for calibration of microdialysis probes procedure was carried out as previous reports (Porkka-Heiskanen et al., 2000; Murillo-Rodríguez et al., 2004; Blanco-Centurión et al., 2006).

#### Histological Verification of Probe Location in Microdialysis Experiments

After all microdialysis experiments, rats were sacrificed with a lethal dose of pentobarbital for the standard procedure for vascular perfusion (saline solution [0.9%] followed by formaldehyde [4%]). Next, the brain was removed and post-fixed overnight in formaldehyde (4%) followed by 10, 20, and 30% sucrose/0.1 M PBS during 24 h (each concentration). At last, all brains were cut in coronal sections (20 μm) using a Portable Bench-top Cryostat (Leica CM1100, Wetzlar, Germany) and collected in 1:5 serial order. One serial was used for probe location and it was identified by plotting using rat brain atlas (Paxinos and Watson, 2005). All histological procedures were developed as previously reported (Murillo-Rodríguez et al., 2004; Blanco-Centurión et al., 2006).

#### Statistical Analysis

As described in Experiment 2.

## Results

### Effects on Sleep After Injections of DZNep or GSK-J1 During the Lights-On or the Lights-Off Period

**FIGURE 1:**
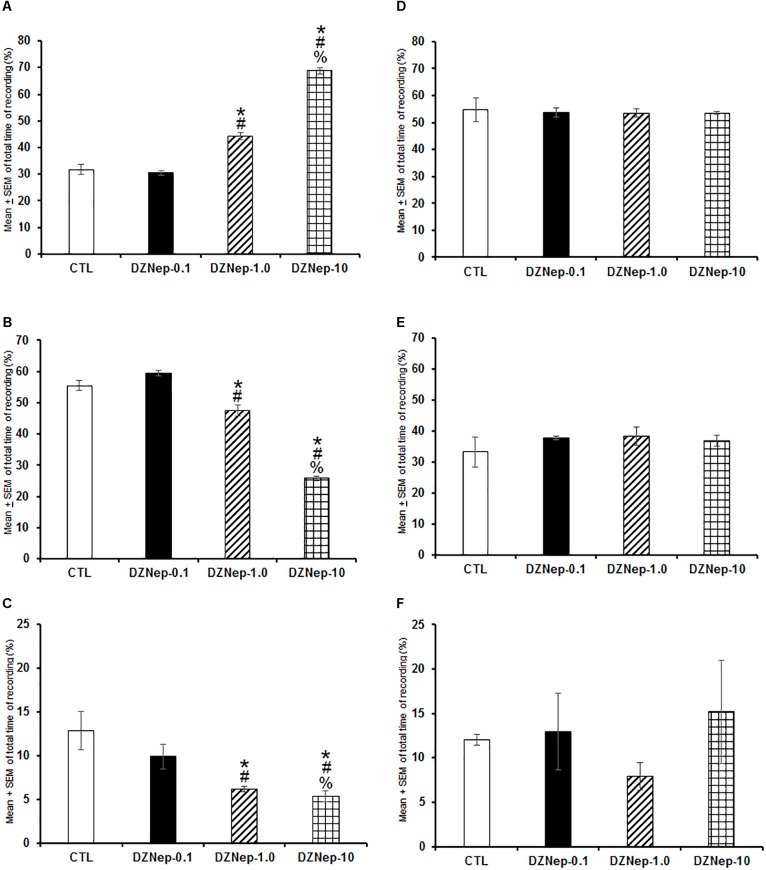
Effects on total time (4 h of sleep recordings) of wakefulness (W), slow wave sleep (SWS), and rapid eye movement sleep (REMS) after injection of vehicle (CTL [*n* = 5]) or DZNep (0.1, 1.0, or 10 mg/kg, i.p.; each dose, *n* = 5) during either lights-on or lights-off period. Effects were observed in W, SWS, or REMS when DZNEp was injected during the lights-on period (**A–C**, respectively; ^∗^ vs. Control; # vs. DZNep-0.1, % vs. DZNep-1.0; *P* < 0.05). However, when administered at the lights-off period, no changes in sleep stages were found (**D–F**, respectively).

In the first experiment, DZNep treatment (0.1, 1.0, or 10 mg/kg, i.p.) given at the beginning of the lights-on period caused a dose-dependent increase of W (Figure 1A; *P* < 0.0001) as well as a decrease in SWS (Figure 1B; *P* < 0.0001) and REMS (Figure 1C; *P* < 0.0001). *Post hoc* analysis test showed significant differences between experimental groups for W, SWS, and REMS (*P* < 0.0001). No statistical changes were observed in total W (Figure 1D; *P* > 0.9), SWS (Figure 1E; *P* > 0.6), or REMS (Figure 1F; *P* > 0.5) if drug was administered at the beginning of the lights-off period. From data obtained, we conclude that waking was enhanced by inhibiting methylase using DZNep if administered during the lights-on period.

**FIGURE 2:**
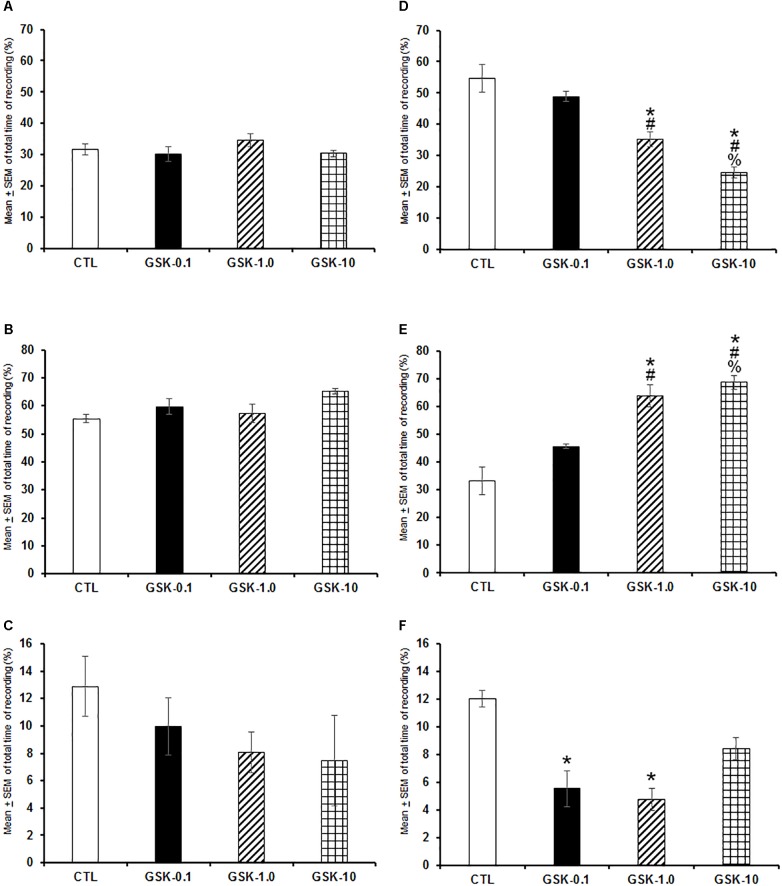
Effects on total time (4 h of sleep recordings) of wakefulness, SWS, and REMS after injection of vehicle (CTL [*n* = 5]) or GSK-J1 (0.1, 1.0, or 10 mg/kg, i.p.; each dose, *n* = 5) during either lights-on or lights-off period. When administered at the lights-on period, no effects in sleep stages were found (**A–C**, respectively). However, if injected during the lights-off period, GSK-J1 decreased W, decreased SWS and diminished REMS (**D–F**, respectively; ^∗^ vs. Control; # vs. GSK-0.1, % vs. GSK-1.0; *P* < 0.05).

When GSK-J1 was injected (0.1, 1.0, or 10 mg/kg, i.p.) at the beginning of the lights-on period, no statistical effects were observed in total time of W (Figure 2A; *P* > 0.3), SWS (Figure 2B; *P* > 0.6), or REMS (Figure 2C; *P* > 0.4). However, if administered at the beginning of the lights-off period, the inhibitor of histone demethylase decreased alertness (Figure 2D; *P* < 0.0001), enhanced SWS (Figure 2E; *P* < 0.0001) and decreased REMS (Figure 2F; *P* < 0.0003). Further *post hoc* analysis test demonstrated significant differences between experimental trials in W, SWS, and REMS (*P* < 0.0001). We conclude that inhibition of histone demethylase promoted sleep-inducing effect when injected during the lights-off period.

### Effects on Sleep Parameters After Administrations of DZNep or GSK-K1 During the Lights-On or the Lights-Off Period

The sleep parameters (Table 1), including the frequency (number of bouts), mean duration (min) and latency (min) of W, SWS, and REMS were analyzed after pharmacological trials following previous procedures (Murillo-Rodríguez et al., 2017). In this regard, administration of DZNep during the lights-on period induced no changes in the number of bouts of waking (*P* > 0.1) whereas SWS (*P* < 0.002) and REMS were decreased (*P* < 0.003). Scheffé’s *post hoc* test showed significant differences between experimental groups for SWS and REMS (*P* < 0.01). In addition, mean duration displayed statistical enhancements in W (*P* < 0.04), with no changes in SWS (*P* > 0.3) but REMS was found increased (*P* < 0.0001). The Scheffé’s *post hoc* test showed us inter-group differences in W and REMS (*P* < 0.01). Lastly, latency for SWS *(P* > 0.3) and REMS (*P* > 0.3) displayed no statistical changes in rats that received DZNep.

**Table 1:** Effects on sleep parameters (frequency, mean duration, and latency) after the administration of vehicle (CTL [*n* = 5]) or DZNep (0.1, 1.0, or 10 mg/kg, i.p.; each dose, *n* = 5) during lights-on period (^∗^ vs. Control; # vs. DZNep-0.1, % vs. DZNep-1.0; *P* < 0.05).

*Wakefulness*	*Frequency (number of bouts)* (*F*_(3,16)_ = 1.849, *P* = 0.1)	*Mean duration (min)* (*F*_(3,16)_ = 3.484, *P* < 0.04)	*Latency (min)*
*CTL*	19.72 ± 1.0	1.70 ± 0.2	0.0 ± 0.0
*DZNep-0.1*	19.48 ± 1.1	1.80 ± 0.1	0.0 ± 0.0
*DZNep-1.0*	24.72 ± 3.6	3.44 ± 1.1	0.0 ± 0.0
*DZNep-10*	16.40 ± 3.1	4.69 ± 0.9 ^∗, %^	0.0 ± 0.0

***Slow wave sleep***	***Frequency (number of bouts)* (*F*_(3,16)_ = 7.804, *P* < 0.002)**	***Mean duration (min)* (*F*_(3,16)_ = 1.168, *P* = 0.3)**	***Latency (min)* (*F*_(3,16)_ = 1.310, *P* = 0.3)**

*CTL*	38.80 ± 1.9	2.96 ± 0.1	26.28 ± 4.0
*DZNep-0.1*	38.08 ± 1.4	3.01 ± 0.2	28.99 ± 4.0
*DZNep-1.0*	37.86 ± 3.8	4.48 ± 1.9	35.28 ± 3.7
*DZNep-10*	23.72 ± 3.0 ^∗, #, %^	5.32 ± 0.7	25.52 ± 4.2

***REMS***	***Frequency (number of bouts)* (*F*_(3,16)_ = 7.092, *P* < 0.003)**	***Mean duration (min)* (*F*_(3,16)_ = 13.946, *P* < 0.0001)**	***Latency (min)* (*F*_(3,16)_ = 1.02, *P* = 0.3)**

*CTL*	19.52 ± 2.3	0.80 ± 0.1	55.32 ± 12.8
*DZNep-0.1*	19.12 ± 2.3	0.80 ± 0.1	50.58 ± 12.8
*DZNep-1.0*	12.0 ± 1.3	1.04 ± 0.3	49.72 ± 7.8
*DZNep-10*	10. 00 ± 0.8 ^∗, %^	2.50 ± 0.2 ^∗, #, %^	32.88 ± 4.0

The analysis of sleep parameters (Table 2) after injection of DZNep during the lights-off period showed no statistical differences among experimental trials in frequency of waking (*P* > 0.5), SWS (*P* > 0.3) and REMS (*P* > 0.2). Mean duration for W, SWS, and REMS showed no significant changes as well (*P* > 0.6, *P* > 0.5, and *P* > 0.9, respectively). Alike results were observed in latency for SWS (*P* > 0.4) and REMS *(P* > 0.4).

**Table 2:** Effects on sleep parameters (frequency, mean duration, and latency) after the injection of vehicle (CTL [*n* = 5]) or GSK-J1 (0.1, 1.0, or 10 mg/kg, i.p.; each dose, *n* = 5) during lights-on period (^∗^ vs. Control; # vs. GSJ-0.1, % vs. GSK-1.0; *P* < 0.05).

*Wakefulness*	*Frequency (number of bouts)* (*F*_(3,16)_ = 0.721, *P* = 0.5)	*Mean duration (min)* (*F*_(3,16)_ = 0.634, *P* = 0.6)	*Latency (min)*
*CTL*	17.70 ± 1.8	5.15 ± 2.9	0.0 ± 0.0
*DZNep-0.1*	23.43 ± 5.5	1.73 ± 0.2	0.0 ± 0.0
*DZNep-1.0*	17.70 ± 1.5	5.53 ± 2.7	0.0 ± 0.0
*DZNep-10*	19.20 ± 1.8	3.91 ± 1.3	0.0 ± 0.0

***Slow wave sleep***	***Frequency (number of bouts)* (*F*_(3,16)_ = 1.186, *P* = 0.3)**	***Mean duration (min)* (*F*_(3,16)_ = 0.675, *P* = 0.5)**	***Latency (min)* (*F*_(3,16)_ = 0.865, *P* = 0.4)**

*CTL*	35.50 ± 1.9	2.38 ± 0.2	28.40 ± 12.6
*DZNep-0.1*	26.53 ± 7.3	2.46 ± 0.3	28.14 ± 1.7
*DZNep-1.0*	32.20 ± 2.1	3.10 ± 0.5	41.28 ± 4.8
*DZNep-10*	35.60 ± 3.9	2.78 ± 0.3	39.52 ± 6.5

***REMS***	***Frequency (number of bouts)* (*F*_(3,16)_ = 1.631, *P* = 0.2)**	***Mean duration (min)* (*F*_(3,16)_ = 0.083, *P* = 0.9)**	***Latency (min)* (*F*_(3,16)_ = 0.991, *P* = 0.4)**

*CTL*	22.68 ± 1.5	0.88 ± 0.08	56.80 ± 12.9
*DZNep-0.1*	11.95 ± 4.0	0.83 ± 0.09	39.98 ± 4.5
*DZNep-1.0*	15.80 ± 2.9	0.80 ± 0.08	51.72 ± 3.7
*DZNep-10*	15.00 ± 4.7	0.80 ± 0.02	41.16 ± 2.1

GSK-J1 caused no statistical differences (Table 3) once injected during the lights-on period in the frequency of waking (*P* > 0.7) but decreased the number of bouts in SWS (*P* < 0.0001) and REMS (*P* < 0.05). The Scheffé’s *post hoc* test showed inter-group differences in SWS and REMS (*P* < 0.005). Mean duration showed no statistical differences in W (*P* > 0.1), SWS *(P* > 0.1), or REMS (*P* > 0.7). Similar findings were observed in latency for SWS (*P* > 0.1) and REMS (*P* > 0.2).

**Table 3:** Effects on sleep parameters (frequency, mean duration, and latency) after the treatment of vehicle (CTL [*n* = 5]) or DZNep (0.1, 1.0, or 10 mg/kg, i.p.; each dose, *n* = 5) during lights-off period (^∗^ vs. Control; # vs. DZNep-0.1, % vs. DZNep-1.0; *P* < 0.05).

*Wakefulness*	*Frequency (number of bouts)* (*F*_(3,16)_ = 0.405, *P* = 0.7)	*Mean duration (min)* (*F*_(3,16)_ = 2.152, *P* = 0.1)	*Latency (min)*
*CTL*	19.72 ± 1.0	1.70 ± 0.2	0.0 ± 0.0
*GSK-0.1*	21.62 ± 1.1	1.71 ± 0.3	0.0 ± 0.0
*GSK-1.0*	21.46 ± 3.7	3.62 ± 1.1	0.0 ± 0.0
*GSK-10*	23.14 ± 1.5	2.59 ± 0.1	0.0 ± 0.0

***Slow wave sleep***	***Frequency (number of bouts)* (*F*_(3,16)_ = 23.793, *P* < 0.0001)**	***Mean duration (min)* (*F*_(3,16)_ = 2.374, *P* = 0.1)**	***Latency (min)* (*F*_(3,16)_ = 1.738, *P* = 0.1)**

*CTL*	38.80 ± 1.9	2.96 ± 0.1	26.28 ± 4.0
*GSK-0.1*	37.86 ± 3.0	2.36 ± 0.2	21.60 ± 4.6
*GSK-1.0*	26.78 ± 3.5 ^∗, #^	7.60 ± 3.7	37.60 ± 9.9
*GSK-10*	13.44 ± 1.0 ^∗, #, %^	8.65 ± 1.8	18.08 ± 5.0

***REMS***	***Frequency (number of bouts)* (*F*_(3,16)_ = 3.238, *P* < 0.05)**	***Mean duration (min)* (*F*_(3,16)_ = 0.476, *P* = 0.7)**	***Latency (min)* (*F*_(3,16)_ = 1.486, *P* = 0.2)**

*CTL*	19.52 ± 2.3	0.80 ± 0.1	55.32 ± 12.8
*GSK-0.1*	20.80 ± 3.2	0.76 ± 0.04	36.92 ± 6.7
*GSK-1.0*	15.40 ± 2.9	0.75 ± 0.07	51.88 ± 13.9
*GSK-10*	10.20 ± 1.9 ^∗, #^	1.17 ± 0.5	27.56 ± 6.9

Table 4 shows the sleep parameters in GSK-J1-treated rats across lights-off period. As demonstrated, no statistical differences were found in frequency for W (*P* > 0.2) while a significant decrease was observed in SWS (*P* < 0.03) as well as REMS (*P* < 0.04). The Scheffé’s *post hoc* test displayed differences for SWS (*P* < 0.008) and REMS (*P* < 0.05). Mean duration showed no statistical changes in alertness (*P* > 0.5) whereas a significant increase was found in SWS (*P* < 0.01) and REMS (*P* < 0.005). The Scheffé’s *post hoc* test showed differences for SWS (*P* < 0.009) and REMS (*P* < 0.01). Finally, latency was found with no significant changes for SWS (*P* > 0.1) but REMS displayed a significant diminution (*P* < 0.01). *Post hoc* test showed significant changes for REMS (*P* < 0.007).

**Table 4:** Effects on sleep parameters (frequency, mean duration, and latency) after the application of vehicle (CTL [*n* = 5]) or GSK-J1 (0.1, 1.0, or 10 mg/kg, i.p.; each dose, *n* = 5) during lights-off period (^∗^ vs. Control; # vs. GSJ-0.1, % vs. GSK-1.0; *P* < 0.05).

*Wakefulness*	*Frequency (number of bouts)* (*F*_(3,16)_ = 1.636, *P* = 0.2)	*Mean duration (min)* (*F*_(3,16)_ = 0.791, *P* = 0.5)	*Latency (min)*
*CTL*	17.70 ± 1.8	5.15 ± 2.9	0.0 ± 0.0
*GSK-0.1*	22.76 ± 3.0	5.05 ± 1.6	0.0 ± 0.0
*GSK-1.0*	27.70 ± 5.8	3.02 ± 1.3	0.0 ± 0.0
*GSK-10*	28.014 ± 3.2	1.81 ± 0.1	0.0 ± 0.0

***Slow wave sleep***	***Frequency (number of bouts)* (*F*_(3,16)_ = 3.608, *P* < 0.03)**	***Mean duration (min)* (*F*_(3,16)_ = 4.436, *P* < 0.01)**	***Latency (min)* (*F*_(3,16)_ = 2.344, *P* = 0.1)**

*CTL*	37.50 ± 1.9	2.38 ± 0.2	28.40 ± 12.3
*GSK-0.1*	34.22 ± 4.3	2.38 ± 0.2	45.04 ± 6.1
*GSK-1.0*	26.52 ± 7.7	12.72 ± 4.4 ^∗, #^	27.00 ± 8.4
*GSK-10*	17.74 ± 1.9 ^∗, %^	9.40 ± 2.2 ^∗, %^	13.88 ± 3.0 ^%^

***REMS***	***Frequency (number of bouts)* (*F*_(3,16)_ = 6.583, *P* < 0.004)**	***Mean duration (min)* (*F*_(3,16)_ = 6.153, *P* < 0.005)**	***Latency (min)* (*F*_(3,16)_ = 4.515, *P* < 0.01)**

*CTL*	22.68 ± 1.5	0.88 ± 0.08	56.80 ± 12.9
*GSK-0.1*	12.00 ± 2.8 ^∗^	0.63 ± 0.1	56.96 ± 6.9
*GSK-1.0*	11.20 ± 1.8 ^∗^	0.50 ± 0.08 ^∗^	34.80 ± 8.9
*GSK-10*	12.60 ± 2.0 ^∗^	1.05 ± 0.1 ^#, %^	18.60 ± 3.5 ^∗, %^

### Effects on Sleep Power Spectra After Treatments of DZNep or GSK-J1 During the Lights-On or the Lights-Off Period

**FIGURE 3:**
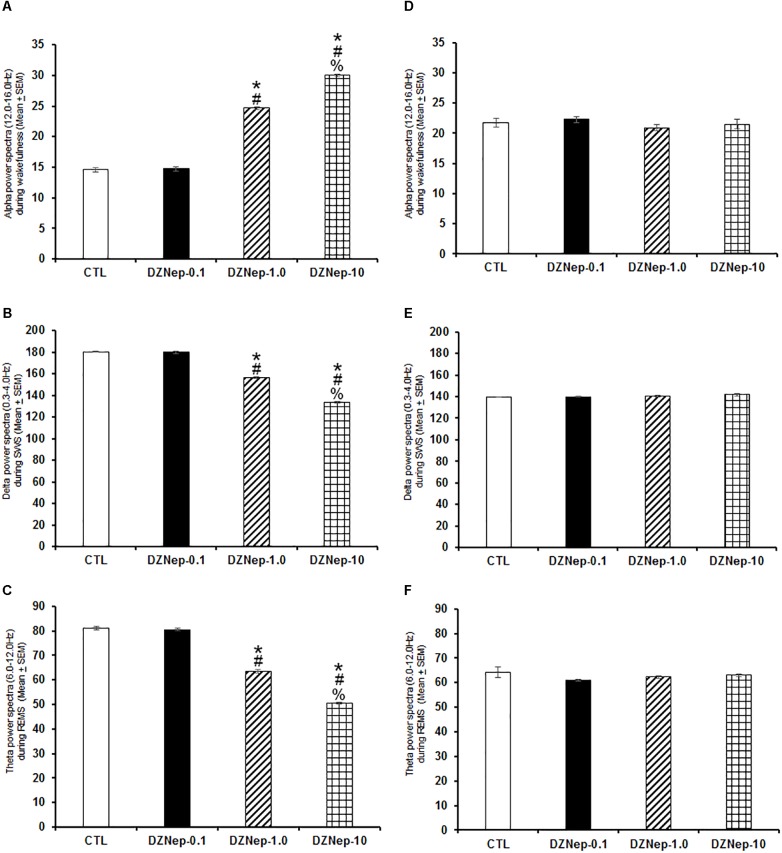
Effects on alpha (during W), delta (across SWS), and theta power spectra (during REMS) after injection of vehicle (CTL [*n* = 5]) or DZNep (0.1, 1.0, or 10 mg/kg, i.p.; each dose, *n* = 5) during either lights-on or lights-off period. Effects were observed in alpha, delta and theta power spectra when DZNEp was injected during the lights-on period (**A–C**, respectively; ^∗^ vs. Control; # vs. DZNep-0.1, % vs. DZNep-1.0; *P* < 0.05). However, when administered at the lights-off period, no changes in power spectra were found (**D–F**, respectively).

Systemic injection of DZNep during lights-on enhanced alpha (Figure 3A; *P* < 0.0001) as well as a decrease in delta (Figure 3B; *P* < 0.0001) and theta power spectra (Figure 3C; *P* < 0.0001). The Scheffé’s *post hoc* test showed inter-group differences for alpha (*P* < 0.0001), delta (*P* < 0.0001), and theta power spectra (*P* < 0.0001). No significant changes were found in power spectra analysis for alpha (Figure 3D; *P* > 0.6), delta (Figure 3E; *P* > 0.6) or theta (Figure 3F; *P* > 0.5) during the lights-off period in DZNep-treated rats.

**FIGURE 4:**
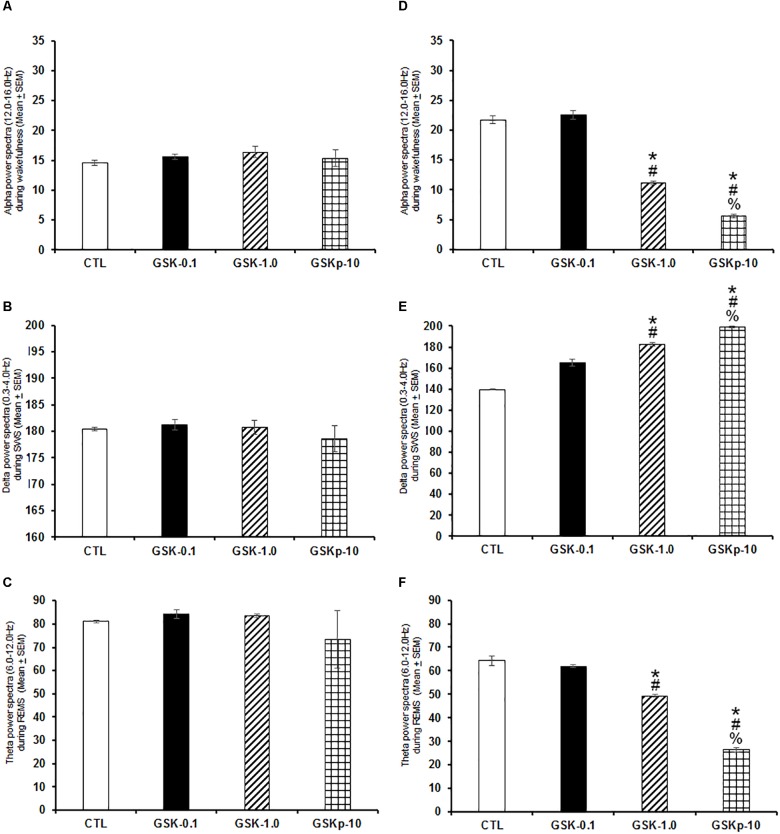
Effects on alpha (during W), delta (across SWS), and theta power spectra (during REMS) after administration of vehicle (CTL [*n* = 5]) or GSK-J1 (0.1, 1.0, or 10 mg/kg, i.p.; each dose, *n* = 5) during either lights-on or lights-off period. When administered at the lights-on period, GSK-J1 caused no statistical changes in alpha, delta or theta power spectra (**A–C**, respectively). However, if injected during the lights-off period, GSK-J1 decreased alpha but decreased delta and diminished theta power spectra (**D–F**, respectively; ^∗^ vs. Control; # vs. GSK-0.1, % vs. GSK-1.0; *P* < 0.05).

Next, we evaluated whether GSK-J1 would induce effects in power spectra if injected during the lights-on or the lights-off phase. Results showed that when administered at the beginning of the lights-on period no statistical changes in alpha (Figure 4A; *P* > 0.6), delta (Figure 4B; *P* > 0.6), or theta power spectra (Figure 4C; *P* > 0.6) were observed. Although if given at the beginning of the lights-off period, the histone demethylation inhibitor diminished alpha (Figure 4D; *P* < 0.0001), but increased SWS (Figure 4E; *P* < 0.0001) and reduced theta power spectra (Figure 4F; *P* < 0.0001). The Scheffé’s *post hoc* test showed inter-group differences for alpha (*P* < 0.0001), delta (*P* < 0.0001), and theta power spectra (*P* < 0.0001).

### Effects on the Extracellular Levels of Monoamines, Adenosine, and Acetylcholine After Administrations of DZNep or GSK-J1

**FIGURE 5:**
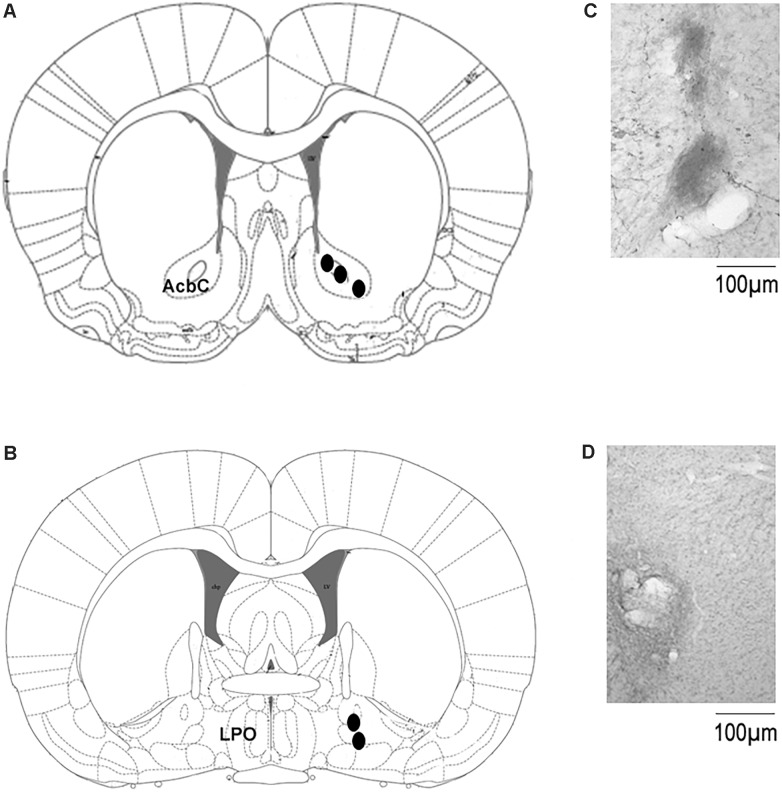
Localization of the microdialysis probe in the microdialysis experiments. **(A,B)** Shows the schematic illustration from the rat brain atlas (Paxinos and Watson, 2005) with the location of the microdialysis probe in AcbC (coordinates: A = +1.2, L = +2.0, and H = –7.0 mm, with reference to Bregma [Paxinos and Watson, 2005]) or basal forebrain (coordinates: A = –0.35; L = 2.0; and H = –7.5 mm, with reference to Bregma [Paxinos and Watson, 2005]), respectively. Microphotographies show the microdialysis probe track in AcbC or basal forebral in **(C,D)**, respectively. Abbreviations were taken from the Paxinos and Watson (2005): AcbC, nucleus accumbens; LPO, lateral preoptic area. Scale bar: 100 μm.

The localization of the microdialysis probe in the AcbC or basal forebrain is shown in Figure 5. The schematic illustration of the position of the probe into ACbC (Figure 5A) or basal forebrain (Figure 5B) is displayed whereas microphotographies from localization of microdialysis cannuale track into ACbC and basal forebrain is also shown (Figure 5C,D, respectively).

**FIGURE 6:**
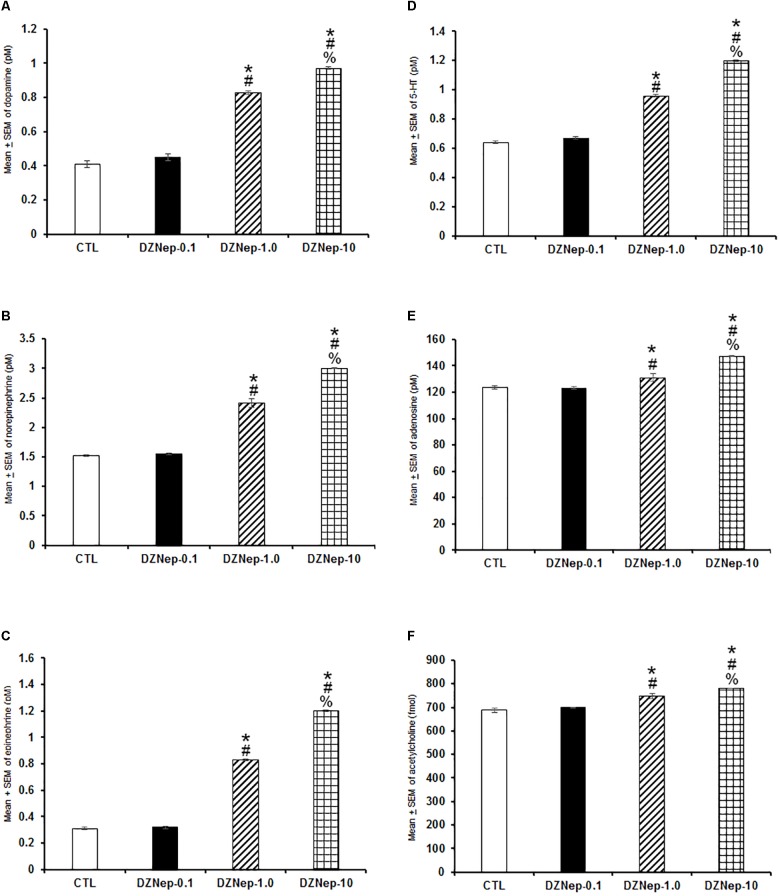
Effects on extracellular levels of dopamine, norepinephrine, epinephrine, 5-HT, adenosine, and acetylcholine (4 h of sampling) after the injection of vehicle (CTL [*n* = 5]) or DZNep (0.1, 1.0, or 10 mg/kg, i.p.; each dose, *n* = 5) during the lights-on period. Effects were observed as an increase in contents of dopamine, norepinephrine, epinephrine, 5-HT, adenosine and acetylcholine after pharmacological challenge (**A–F**, respectively; ^∗^ vs. Control; # vs. DZNep-0.1, % vs. DZNep-1.0; *P* < 0.05).

Since statistical changes in DZNep-treated rats were observed in sleep studies during the lights-on period (see section “Results”), our next aim was to determinate whether administrations of this compound would promote neurochemical changes if injected only at the beginning of the lights-on period. Having this aim in mind, we injected DZNep (0.1, 1.0, or 10 mg/kg, i.p.) to rats and collected samples from microdialysis probes during the lights-on phase. Injection of compound caused a dose-dependent enhancement in extracellular contents of DA (Figure 6A; *P* < 0.0001), NE (Figure 6B; *P* < 0.0001), EP (Figure 6C; *P* < 0.0001), 5-HT (Figure 6D; *P* < 0.0001), AD (Figure 6E; *P* < 0.0001), and ACh (Figure 6F; *P* < 0.0001). The Scheffé’s *post hoc* test showed inter-group significant differences for DA (*P* < 0.0001), NE (*P* < 0.0001), EP (*P* < 0.0001), 5-HT (*P* < 0.0001), AD (*P* < 0.0001), and ACh (*P* < 0.03). We conclude that DZNep was able to increase several sleep-related neurochemicals when injected during the lights-on period.

**FIGURE 7:**
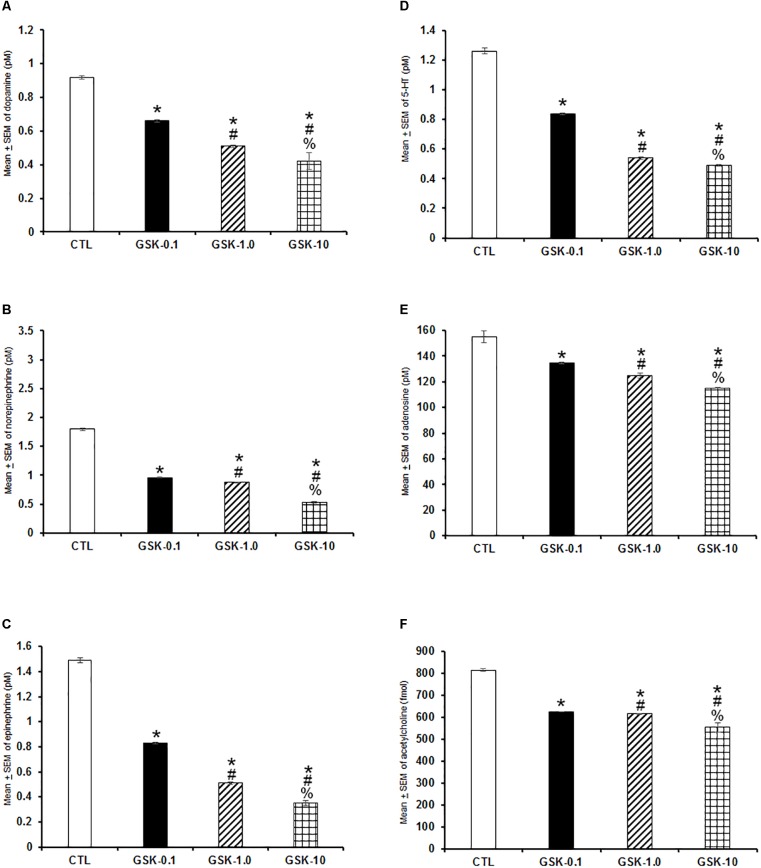
Effects on extracellular levels of dopamine, norepinephrine, epinephrine, 5-HT, adenosine, and acetylcholine (4 h of sampling) after the administration of vehicle (CTL [*n* = 5]) or GSK-J1 (0.1, 1.0, or 10 mg/kg, i.p.; each dose, *n* = 5) during the lights-off period. The levels of dopamine, norepinephrine, epinephrine, 5-HT, adenosine, and acetylcholine were found decreased after pharmacological trials (**A–F**, respectively; ^∗^ vs. Control; # vs. GSK-0.1, % vs. GSK-1.0; *P* < 0.05).

In the following experiment, we evaluated if GSK-J1 would be able to provoke effects in extracellular levels of DA, NE, EP, 5-HT, AD, and ACh. Due that GSK-J1 exerted influence on sleep during the lights-off period, we administered this compound (0.1, 1.0, or 10 mg/kg, i.p.) at the beginning of the dark period. A decrease in a dose-dependent fashion of the contents of DA (Figure 7A; *P* < 0.0001), NE (Figure 7B; *P* < 0.0001), EP (Figure 7C; *P* < 0.0001), 5-HT (Figure 7D; *P* < 0.0001), AD (Figure 7E; *P* < 0.0001), and ACh (Figure 7F; *P* < 0.0001) was found. The Scheffé’s *post hoc* test showed inter-group significant differences for DA (*P* < 0.0001), NE (*P* < 0.0001), EP (*P* < 0.0001), 5-HT (*P* < 0.0001), AD (*P* < 0.0004), and ACh (*P* < 0.0001). In conclusion, GSK-J1 diminished the levels of neurochemicals if administered during the lights-off period.

## Discussion

Histone methylation/demethylation activity has been studied in several biomedical aspects, with emphasis in cancer (Kunts et al., 2017; Pérez et al., 2018; Porcellini et al., 2018; Tong et al., 2018; Wijenayake et al., 2018). Despite significant advances have been achieved by studying the molecular role of histones in regulating health conditions, no direct evidence was available regarding the influence of histone methylation/demethylation activity on neurobiological phenomena such as the sleep-wake cycle and sleep-related neurochemicals. Here, we described that histone methylation inhibition by DZNep enhanced waking if injected during the lights-on period whereas histone demethylation inhibition by GSK-J1 caused a sleep-inducing effect if administered during the lights-off period. Further effects were observed in DZNep-treated rats in sleep parameters during the lights-on period. In addition, animals that received GSK-J1 showed changes in sleep parameters only if injected during the lights-off period. Moreover, animals that received DZNep showed across the lights-on period an increase in alpha power spectra whereas delta and theta were found increased, in SWS and REMS, respectively. Opposite findings were observed in GSK-J1-treated animals during the lights-off since they displayed a significant enhancement in alpha power spectra as well as a decrease in delta and theta power spectra, during SWS and REMS, respectively. In addition, the histone methylation activity also induced neurochemical changes. For instance, DZNep injection at the beginning of the lights-on period caused a dose-dependent enhancement in extracellular contents of DA, NE, EP, 5-HT, AD, and ACh whereas GSK-J1 administered at the beginning of the lights-off period, decreased the levels of the neurochemicals mentioned above. We conclude that effects observed after the administration of the drugs during the light or dark phase suggest a possible sensitivity of the availability of histone methylation or demethylation depending on the phase of the light–dark cycle. This possible sensitivity could be the cause of the effects observed in sleep and neurochemical levels.

Although current findings provide novel insights of the likely role of inhibition of either histone methylation or histone demethylation on sleep and neurochemicals modulation, several limitations in the current study have been identified. For example, it is difficult to determine whether the changes observed in sleep may be cause or effect on the variations found in the neurochemicals analyzed. Moreover, previous reports have indicated that DZNep globally inhibits histone methylation acting as a non-selective molecular drug (Miranda et al., 2009). Thus, experiments testing the effects on sleep by using selective drugs should be design in the near future. Furthermore, the compounds were administered by systemic route raising the possibility that central injections (i.c.v. or into parenchyma) might provoke different results. Future studies using histone methylation/demethylation inhibitors may target brain areas related to the control of the sleep-wake cycle such as thalamus, hypothalamus or pedunculopontine tegmentum nucleus.

Novel therapies aimed to treat cancer by using histone methylation drugs show positive results (Harb-de la Rosa et al., 2015; Pleyer and Greil, 2015; Castilho et al., 2017; Choi et al., 2017; Curry et al., 2018). It would be interesting to determine whether using DZNep may control sleepiness in patients with cancer or if GSK-J1 might modulate insomnia caused by stress prior to undergoing radiotherapy (Davies et al., 2017; Vin-Raviv et al., 2017; Miladinia et al., 2018; Zhou et al., 2018). Testing this hypothesis in experimental models of sleep disturbances would give us an important comprehensive insight of the role of histone methylation/demethylation in the management of sleep disturbances. Moreover, circadian fluctuations of histone methylation/demethylation activity would provide us foundations for in which ‘time’ becomes a critical variable to obtain the desirable effects. Thus, chronopharmacological studies would embrace endogenous periodicities and biological timing of the activity of histones to ensure that drugs are acting on time coupled with these rhythmicities and, then enhance the expected effects.

Although limited evidence is available in regards the role of histone methylation/demethylation activity in neurobiological phenomena, our studies extend these data to suggest that nuclear elements could induce sleep and neurochemical modulation (Duan et al., 2016; Gaine et al., 2018; Morales-Lara et al., 2018; Shalaby et al., 2018). Following this idea, we have developed a conceptual framework that suggests that manipulation of histone methylation/demethylation activity could engage sleep-related genes: chromatin organization by histone methylation/demethylation, implies the modulation of the activity of large numbers of genes (MacPherson et al., 2018; Lamadema et al., 2019; Ricketts et al., 2019), including those that have been associated with sleep control (Wang and Liu, 2016; Tomita et al., 2017; Kim et al., 2018). However, the role of histone methylation/demethylation in sleep remains poorly understood. Therefore, this is the first direct evidence that drugs aimed to inhibits histone methylation or demethylation are related with sleep and neurochemicals effects. In addition, the importance of DNA-methylation and demethylation in neurotransmission has been partially studied. Despite that several studies have reported the link between methylation or demethylation effects on several neurobiological processes, such as learning and memory as well as pathological conditions, it is difficult to draw solid conclusions about how the histone methylation and demethylation inhibition may exert influence on the neurotransmitter contents. The available data regarding the modifications of histones in conjunction with behavior, implicitly suggest changes in neurotransmission (Sun et al., 2013; Cholewa-Waclaw et al., 2016; Bayraktar and Kreutz, 2018). With respect to those results seen with relationship between behavioral functions and histone activity, it is conceivable that perhaps those effects could be attributable to neurotransmitters alterations. However, it remains to be described if histone methylation and demethylation inhibition induce changes in neurotransmitters by engaging enzymatic process involved in the formation of DA, NE, EP, 5-HT, AD, and ACh. It cannot be rule out that enzymatic component in the metabolic pathway of studied neurotransmitters could be inhibited by the methylation or demethylation inhibition. Importantly, the DNA methylation and histone acetylation have been implicated in the modulation of synaptic transmission of several neurotransmitters, including key genes in the GABAergic pathway (Nelson and Monteggia, 2011; Holloway and González-Maeso, 2015; Shrestha and Offer, 2016). Indeed, complementary experiments testing the role of methylation and demethylation inhibition on the biosynthesis of DA, NE, EP, 5-HT, AD, and ACh are needed to provide better understanding of the phenomena. Further studies will be required to determine the specific mechanism of action of DZNep or GSK-J1 on sleep and neurochemical modulation. We assume that in the near future, additional studies might describe the histone methylation or demethylation profiles as molecular markers for diagnostics of sleep disturbances or the application of drugs such as DZNep or GSK-J1 at specific time of the circadian rhythm as therapeutic strategy. Taken together, our findings demonstrate that histone methylation inhibition promotes waking whereas sleep is promoted when histone demethylation is suppressed.

## Data Availability

The datasets generated for this study are available on request to the corresponding author.

## Author Contributions

EM-R designed and implemented experiments, analyzed the data, assembled the figures, and edited the manuscript. GA-S contributed with technical EEG/EMG surgical support. JAB edited the manuscript and revised statistical analyses. NBR helped with revision of experimental designs. TY provided statistical analysis guidance. SM contributed with revision of literature and experimental designs. HB provided guidance with writing the manuscript. DT-C revised data and supported assembling the figures. DM contributed editing statistical data. LC contributed revising data and editing the manuscript. ABV contributed editing and analyzing data. All authors revised all data set and approved the final version of the manuscript.

## Conflict of Interest Statement

The authors declare that the research was conducted in the absence of any commercial or financial relationships that could be construed as a potential conflict of interest.
